# Agnosia for head tremor in essential tremor: prevalence and clinical correlates

**DOI:** 10.1186/s40734-016-0032-0

**Published:** 2016-02-12

**Authors:** Hatice N. Eken, Elan D. Louis

**Affiliations:** Department of Neurology, Yale School of Medicine, Yale University, LCI 710, 15 York Street, PO Box 208018, New Haven, CT 06520-8018 USA; Department of Chronic Disease Epidemiology, Yale School of Public Health, Yale University, New Haven, CT USA; Center for Neuroepidemiology and Clinical Neurological Research, Yale School of Medicine, Yale University, New Haven, CT USA

**Keywords:** Essential tremor, Clinical, Head tremor, Agnosia

## Abstract

**Background:**

Lack of awareness of involuntary movements is a curious phenomenon in patients with certain movement disorders. An interesting anecdotal observation is that patients with essential tremor (ET) often seem unaware of their own head tremor. In the current study, we asked ET patients whether they were aware of head tremor while it was occurring on examination, thereby allowing us to gauge real-time awareness of their involuntary movement.

**Methods:**

ET cases enrolled in an ongoing clinical research study at the Columbia University Medical Center (2009–2014). During a videotaped tremor examination, they were questioned about the presence of head tremor. True positives were cases who exhibited head tremor on examination and were aware of it; false negatives were cases who exhibited head tremor but were unaware of it.

**Results:**

The 126 ET cases had a mean age of 72.6 ± 12.4 years. Nineteen (48.7 %) of 39 cases with head tremor on examination did not report having head tremor at that moment. Even among cases with moderate or severe head tremor on examination, unawareness of head tremor was 45.5 %. We assessed the clinical correlates of unawareness of head tremor, comparing true positives to false negatives, and unawareness was correlated with older age, lower mental status test scores and several other clinical variables.

**Conclusions:**

Nearly one-half of ET cases with head tremor on examination were acutely unaware of their tremor. Whether such agnosia for tremor may be leveraged as a diagnostic feature of ET is a question for future clinical studies.

**Electronic supplementary material:**

The online version of this article (doi:10.1186/s40734-016-0032-0) contains supplementary material, which is available to authorized users.

## Background

Lack of awareness of involuntary movements is a fascinating and not infrequently observed phenomenon in patients with certain movement disorders. As such, patients with Parkinson’s disease are often unaware of their levodopa-induced dyskinesias and patients with Huntington’s disease are often not aware of chorea [1, 2]. An interesting anecdotal observation is that essential tremor (ET) patients often seem unaware of their head tremor. In a prior study, we showed that one-third to one-half of ET cases who were observed to have head tremor on neurological examination had not responded “yes” to the question “do you sometimes have head tremor”, which had been asked of them during a previously-administered questionnaire [3]. However, in that study, awareness of head tremor was not assessed in real time (i.e., at the precise instant that tremor was observed on neurological examination); hence, there was a disconnect between the timing of the questionnaire and the timing of the examination. We prospectively designed the current study with this in mind. We asked ET patients whether they were aware of their head tremor while it was occurring on examination, thereby allowing us to gauge real-time awareness of their involuntary movement. In addition to assessing the prevalence of unawareness, we also assessed a broad range of clinical features that we hypothesized could track with greater vs. lesser awareness. The overall goal was to improve our understanding of the clinical phenomenology in ET. As the presence and features of head tremor are often at the center of diagnostic difficulties in ET (e.g., distinguishing ET from dystonia) [4], it is possible that additional clinical insights could lead to additional diagnostic tools and improved diagnostic accuracy.

## Methods

ET cases were enrolled in an ongoing research study of the environmental epidemiology of ET at Columbia University Medical Center (CUMC; 2009–2014) [5]. Cases were derived from two sources: (1) a computerized billing database of ET patients at the Neurological Institute of New York, CUMC, and (2) advertisements to members of the International Essential Tremor Foundation. Cases lived within two hours driving distance of CUMC in New York, New Jersey, and Connecticut. Prior to enrollment, all cases received a diagnosis of ET from their treating neurologist. The CUMC Internal Review Board approved all study procedures, and written informed consent was obtained upon enrollment. Analysis of data was also approved by the Internal Review Board at Yale Medical School.

After enrollment, all ET cases underwent a detailed in-person evaluation conducted by trained research staff, which included the collection of demographic (e.g., age, gender, ethnicity, education) and medical history information. They were asked as variety of questions about their tremor, including, “Does your tremor embarrass you?”, “Does your voice almost always tremble when you talk?”, “Do you ever have an internal sensation that you are having a head tremor?” and “When you feel your head shaking, do you ever see it in the mirror”? The Center for Epidemiological Studies Depression Scale (CES-D) [6], a self-report ten-item screening questionnaire was used to assess depressive symptoms (range = 0 – 30 [greater depressive symptoms]). The Folstein Mini Mental Status Examination (range = 0 – 30) [7] was used to briefly assess cognition.

A videotaped tremor examination included assessments of arm, head (i.e., neck) and voice tremors. During the assessment of neck tremor, the neck muscles were exposed in order to facilitate the evaluation of any dystonic spasms and/or neck muscle hypertrophy. During the videotape, ET cases were asked “Are you experiencing any head tremor at the moment?” This question was asked at two time points (Time point 1 and Time point 2) when the videographer observed head tremor and at one time point when no head tremor was observed by the videographer.

All videotaped neurological examinations were reviewed by a senior neurologist specializing in movement disorders (E.D.L.). Neck tremor in ET was coded as present or absent and was distinguished from dystonic tremor by the absence of twisting or tilting movements of the neck, jerk-like or sustained neck deviation, or hypertrophy of neck muscles. Head tremor had to be both rhythmic and oscillatory to be ascribed to ET. The severity of the head tremor was assessed using a 0 – 3 scale (0 = absent, 1 = mild, 2 = moderate, 3 = severe) [8]. The total tremor score (range = 0 – 36) was the sum of all 0 – 3 postural and kinetic tremor ratings in the arms [5].

Using the clinical questionnaire and videotape data, the diagnosis of ET was reconfirmed in 126 ET cases using published diagnostic criteria (either moderate or greater amplitude kinetic tremor during at least three activities, or a head tremor in the absence of Parkinson’s disease, dystonia, or another neurological disorder) [9].

Awareness of head tremor was indicated using four different groups. *True positives* were cases who exhibited head tremor on examination and who were aware of that tremor when questioned. *True negatives* were cases who did not exhibit head tremor on examination and indicated that they were not experiencing tremor when questioned. *False positives* were cases who did not exhibit head tremor on examination but reported its presence when asked. *False negatives* were patients who exhibited head tremor on examination but were unaware of it. *Unawareness of head tremor* was the number of cases who reported no head tremor over the number of cases who had head tremor on examination (i.e., false negatives/false negatives + true positives). We report unawareness separately at Time points 1 and 2.

We assessed the clinical correlates of awareness of head tremor, comparing true positives to false negatives (Table 1). We carefully chose a range of variables for which we had an *a priori* hypothesis about an association with awareness (e.g., longer duration of tremor and higher education might be associated with greater awareness; similarly being in the workforce might be associated with greater awareness) (Table 1). Given the potential for learning effects by Time 2 (i.e., cueing the patient with a reduction in the number of false negatives), we focused these analyses on Time point 1. These analyses were performed using the statistical software package SPSS (version 21.0; SPSS, Inc., Chicago, Ill., USA). All tests were 2-sided, and significance was accepted at the 5 % level. Using a Kolmogorov-Smirnov test, we determined that several continuous variables were not normally distributed. For these, we compared group differences using a nonparametric (Mann–Whitney U) test rather than a Student’s t test. Chi-square (χ^2^) tests or Fisher’s Exact tests were used to compare categorical variables.

**Table 1:** Clinical characteristics of participants

	All participants	True positives^d^	False negatives^e^	p value*
n	126	20	19	
Age (years)	72.6 ± 12.4	73.5 ± 15.0	81.5 ± 6.9	0.04^a^
Female gender	64 (50.8)	15 (75.0)	12 (63.2)	0.50^b^
White ethnicity	117 (92.9)	19 (95.0)	16 (84.2)	0.34^b^
Current smoker	6 (4.8)	0 (0.0)	1 (5.3)	0.49^b^
Education (years)	15.8 ± 2.8	15.6 ± 2.9	14.9 ± 2.5	0.44^a^
Married	69 (54.8)	7 (35.0)	6 (31.6)	0.86^b^
Lives alone	42 (33.3)	12 (60.0)	9 (47.4)	0.43^b^
Currently in work force	26 (20.6)	3 (15.0)	1 (5.3)	0.61^b^
Years since last hospitalization	10.7 ± 14.5 [4.0]	7.6 ± 12.1 [3.0]	12.8 ± 21.8 [4.0]	0.36^c^
Answered “yes” to the question “Does anyone in your family have tremor?”	83 (65.9)	14 (70.0)	10 (52.6)	0.27^b^
Age first noticed tremor (years)	39.5 ± 20.1	41.3 ± 21.5	41.4 ± 16.9	0.98^a^
Duration of tremor (years)	33.2 ± 19.1	32.3 ± 21.9	40.0 ± 17.7	0.25^a^
Answered “yes” to the question “Does your tremor embarrass you?”	67 (53.2)	11 (55.0)	12 (63.2)	0.61^b^
Answered “yes” to “Does your voice almost always tremble when you talk?”	26 (20.6)	9 (50.0)	4 (25.0)	0.13^b^
Reply to “Do you ever have an internal sensation that you are having a head tremor?”				0.016^b^
No	84 (66.7)	5 (25.0)	12 (63.2)	
Yes	42 (33.3)	15 (75.0)	7 (36.8)	
Reply to “When you feel your head shaking, do you ever see it in the mirror”?				0.002^b^
No	87 (69.0)	3 (15.0)	12 (63.2)	
Yes	39 (31.0)	17 (85.0)	7 (36.8)	
Reply to “How often do you feel nervous or anxious?”				0.36^b^
Never/No	48 (38.1)	6 (30.0)	8 (42.1)	
Rarely	17 (13.5)	4 (20.0)	2 (10.5)	
Sometimes	34 (27.0)	6 (30.0)	7 (36.8)	
Frequently	16 (12.7)	1 (5.0)	2 (10.5)	
Always/Yes	11 (8.7)	3 (15.0)	0 (0.0)	
CESD score	9.0 ± 5.7	8.8 ± 5.0	9.0 ± 5.8	0.91^a^
Severity rating of head tremor (examination)^f^				0.81^b^
1 (mild)	17 (13.5)	8 (40.0)	9 (47.4)	
2 (moderate)	19 (15.1)	10 (50.0)	9 (47.4)	
3 (severe)	3 (2.4)	2 (10.0)	1 (5.3)	
Total tremor score (examination)	21.1 ± 6.2	23.9 ± 6.5	21.05 ± 6.7	0.19^a^
Folstein Mini Mental Status Examination Score	28.4 ± 1.9 [29.0]	29.0 ± 1.6 [30.0]	27.6 ± 1.9 [28.0]	0.01^c^

All values represent means ± standard deviations [median] or numbers (percentage)

Center for Epidemiological Studies Depression Scale

*Comparing true positives vs. false negatives

^a^Student’s t test

^b^Chi square test or Fisher’s Exact test

^c^Mann Whitney test

^d^True positives were cases who exhibited head tremor on examination and who were aware of that tremor when questioned

^e^False negatives were patients who exhibited head tremor on examination but were unaware of it

^f^Based on 39 ET cases with head tremor on examination at Time point 1

## Results

The 126 ET cases had a mean age of 72.6 ± 12.4 years. Nineteen of 39 cases with head tremor on examination at Time point 1 did not report head tremor; i.e., unawareness of head tremor at Time point 1 was 48.7 % (Additional file 1). Even among 22 cases with moderate or severe head tremor on examination, 10 (45.5 %) were unaware of head tremor (Table 1, Additional file 1). At time point 2, unawareness of head tremor was approximately 10 % less (13/34 = 38.2 %).

We assessed the correlates of awareness, comparing true positives to false negatives (Table 1). True positives were nearly a decade younger than false negatives (*p* = 0.04) and they had higher Folstein Mini Mental Status Examination scores (*p* = 0.01) (Table 1). A larger proportion of true positives than false negatives answered “yes” to the questions “Do you ever have an internal sensation that you are having a head tremor?” (*p* = 0.016) and “When you feel your head shaking, do you ever see it in the mirror?” (*p* = 0.002) (Table 1). A larger percentage of true positives than false negatives were female (75.0 % vs. 63.2 %) and white (95.0 % vs. 84.2 %), were currently in the work force (15.0 % vs. 5.3 %), reported a family history of tremor (70.0 % vs. 52.6 %), and answered “yes” to the question “Does your voice almost always tremble when you talk?” (50.0 % vs. 25.0 %), but not to a statistically significant degree (Table 1). Interestingly, the severity of head tremor on examination was not significantly associated with greater awareness (*p* = 0.81), although two of three ET cases with severe head tremor on examination were aware of it (Table 1).

## Discussion

Nearly one-half of ET cases with head tremor on examination were acutely unaware of their tremor. Even among cases with moderate or severe head tremor on examination, unawareness of head tremor was similarly high (45.5 %). In a prior study that used a different design [3], we reported a similar result; however, in that study we did not link the question about head tremor with the observation of head tremor on examination. The current study makes the point that even when asked about tremor at the moment of tremor, one-half of the ET cases were unaware of it.

Agnosia of head tremor could be the result of changes in the regions of the brain that perceive head tremor. Perhaps the brain develops mechanisms to shut down the stimuli caused by these involuntary movements. With some types of oscillatory cranial movements (e.g., patients with congenital nystagmus, who rarely experience oscillopsia), perceptual stability (i.e., the lack of awareness of nystagmus) is achieved through a reduced sensitivity to the motion [3]. Development of such mechanisms in the brain might have had an evolutionary advantage, as it would have allowed individuals to focus their attention on external stimuli that were novel to them, instead of being distracted by a repetitive movement.

The clinical correlates of awareness of head tremor have not been assessed previously in any study. We carefully chose a range of variables for which there was an *a priori* hypothesis about an association with awareness (e.g., longer duration of tremor and higher education might be associated with greater awareness). Cases who were unaware of their head tremor were almost a decade older than those who were aware, and they had lower scores on the Folstein Mini Mental Status Examination (Table 1). This might indicate that unawareness might be a result of cognitive deterioration. Our results were not, however, confounded by the presence of dementia. In a secondary analysis, we assessed the number of cases with Folstein Mini Mental Status Examination scores below <25, an indicator of dementia [10]. We found that only 2 of the cases (one true positive and one false negative) had such scores. A number of other factors correlated with awareness, but not to a significant degree, including education and whether the cases was currently in the work force. Significantly more of the true positives answered “yes” to the questions “Do you ever have an internal sensation that you are having a head tremor?” and “When you feel your head shaking, do you ever see it in the mirror?” This confirms a greater self-awareness of head tremor in these individuals.

This study assessed awareness of tremor at a precise instant in time. Questionnaires that assess whether tremor is more chronically present (e.g., “do you sometimes have tremor”) could result in more true positives. Indeed, in our prior study [3], which asked patients in a similar setting whether they sometimes had tremor, unawareness of head tremor was only 38.7 %, compared to 48.7 % here.

A limitation of this study is that our sample size is relatively small. Although the number of cases studied was 126, there only 39 cases who were true positives or false negatives. As such, some of the associations with non-significant p values could be significant in a larger sample. Hence, they were reported here.

## Conclusions

In summary, nearly one-half of ET cases with head tremor on examination were acutely unaware of their tremor. Whether such agnosia for tremor can be leveraged as a diagnostic feature of ET is a question for future studies.

## Ethics approval and consent to participate

The CUMC Internal Review Board approved all study procedures, and written informed consent was obtained upon enrollment. Analysis of data was also approved by the Internal Review Board at Yale Medical School. The four patients shown in the videotapes signed informed consent for the publication of their videotapes for educational purposes.

## Additional file

Additional file 1:
**Videotapes of 4 patients.** Patient 1: The patient, a 76 year old man with a 28 year history of ET, has moderate horizontal head tremor; he is unaware of it when questioned. Patient 2: The patient, a 73 year old woman with a 32 year history of ET, has mild horizontal head tremor; she is unaware of it when questioned. Patient 3: The patient, a 70 year old woman with a 29 year history of ET, has mild horizontal head tremor; she is unaware of it when questioned. Patient 4: The patient, an 84 year old man with a 23 year history of ET, has moderate head tremor that occurs mainly in the vertical plane; he is unaware of it when questioned. It is important to note that the videographer is positioned to the patient’s right in each case; several of the patients (esp. 1 and 4) have turned tilted their heads to accommodate. When the videographer moves to the left side, the patients turn or tilt their heads in that direction to accommodate, indicating that their head positioning is not dystonic but is voluntary. Complete videotaped neurological examination in each case does not show any torticollis or other signs of dystonia. (MP4 8521 kb)

## Abbreviations

ET: Essential tremor
CUMC: Columbia University Medical Center
CES-D: Center for epidemiological studies depression scale

## References

[1] Vitale C, Pellecchia MT, Grossi D, Fragassi N, Cuomo T, Di Maio L (2001). Unawareness of dyskinesias in Parkinson’s and Huntington’s diseases. Neurol Sci.

[2] Snowden JS, Craufurd D, Griffiths HL, Neary D (1998). Awareness of involuntary movements in Huntington disease. Arch Neurol.

[3] Louis ED, Pellegrino KM, Rios E (2008). Unawareness of head tremor in essential tremor: a study of three samples of essential tremor patients. Mov Disord.

[4] Jain S, Lo SE, Louis ED (2006). Common misdiagnosis of a common neurological disorder: how are we misdiagnosing essential tremor?. Arch Neurol.

[5] Louis ED, Jiang W, Gerbin M, Viner AS, Factor-Litvak P, Zheng W (2012). Blood harmane (1-methyl-9H-pyrido[3,4-b]indole) concentrations in essential tremor: repeat observation in cases and controls in New York. J Toxicol Environ Health A.

[6] Andresen EM, Malmgren JA, Carter WB, Patrick DL (1994). Screening for depression in well older adults: evaluation of a short form of the CES-D (Center for Epidemiologic Studies Depression Scale). Am J Prev Med.

[7] Folstein MF, Folstein SE, McHugh PR (1975). “Mini-mental state”: a practical method for grading the cognitive state of patients for the clinician. J Psychiatr Res.

[8] Herndon RM (1997). Handbook of neurologic rating scales.

[9] Louis ED, Ottman R, Ford B, Pullman S, Martinez M, Fahn S (1997). The Washington Heights-Inwood genetic study of essential tremor: methodologic issues in essential tremor research. Neuroepidemiology.

[10] Roalf DR, Moberg PJ, Xie SX, Wolk DA (2013). Comparative accuracies of two common screening instruments for classification of Alzheimer’s disease, mild cognitive impairment, and healthy aging. Alzheimers Dement.

